# Exploring the difference between self-harm and suicidal ideation among older people who present to emergency departments: national cohort study

**DOI:** 10.1192/bjo.2026.12112

**Published:** 2026-10-15

**Authors:** Fiona Hoare, Geraldine McCarthy, Katerina Kavalidou, Vincent Russell, Michael John Norton, Anne M. Doherty

**Affiliations:** Department of Liaison Psychiatry, Mater Misericordiae University Hospital, Dublin, Ireland; Department of Psychiatry, University of Galway College of Medicine Nursing and Health Sciences, Galway, Ireland; Psychiatry of Later Life, Sligo University Hospital, Sligo, Ireland; WHO Collaborating Centre for Surveillance and Research in Suicide Prevention, National Suicide Research Foundation, Cork, Ireland; HSE, National Clinical Programme for Self-harm and Suicide-related Ideation, Dublin, Ireland; Royal College of Surgeons in Ireland, Dublin, Ireland; Office of Mental Health Engagement and Recovery, Health Service Executive, Dublin, Ireland; School of Applied Social Studies, University College Cork, Dublin, Ireland; Department of Psychiatry, University College Dublin School of Medicine, Dublin, Ireland

**Keywords:** Self-harm, suicide, suicidal ideation, gerontology, emergency treatment

## Abstract

**Background:**

Older people have higher rates of death by suicide, and both self-harm and suicidal ideation are major risk factors for suicide. There is limited data relating to this group internationally.

**Aims:**

The objective of this study was to identify the clinical and sociodemographic variables associated with psychiatric admissions for older people who present with self-harm and suicidal ideation to emergency departments in Ireland.

**Method:**

This is a cohort study of older people in Ireland, utilising data collected by the National Clinical Programme for Self-Harm and Suicide-Related Ideation (NCPSHI). It is a national data-set including anonymised data from all participating emergency departments in Ireland, comprising 72 810 presentations over 5 years (2018–2020). We examined the characteristics of people aged over 60 presenting with self-harm and suicidal ideation (*n* = 4866). The variables associated with suicidal ideation and behaviours were explored using multivariable analysis.

**Results:**

Nationally, 7% of the patients presenting were aged over 60 years. One-quarter (26.8%) of older people presenting with self-harm required psychiatric admission (16.3% all-ages). On multivariable analysis, the relationship between self-harm and psychiatric admission was not statistically significant (reference group was those with suicidal ideation), after controlling for gender, year of presentation, substance use and need for medical admission odds ratio 0.977 (CI0.936–1.020).

**Conclusions:**

Our results outline the characteristics of older people presenting to Irish emergency departments with self-harm and suicidal ideation, which are associated with psychiatric admission. Further research is needed to understand the factors driving these presentations and to tailor our services to meet the needs of older people.

Self-harm and suicide are major public health concerns. Self-harm is defined by the NICE guidelines as ‘intentional self-poisoning or injury, irrespective of the apparent purpose.’^1^ This may include acts with or without suicidal intent and those with some degree of ambivalence. While self-harm may serve a variety of functions for the person, it is established as a significant risk factor for future death by suicide.^2,3^ In older people, self-harm is associated with a 20-fold to 67-fold increase in the odds of an unnatural death compared with a comparison group.^4,5^ Suicidal ideation is associated with elevated rates of suicide, up to 10-fold compared with population rates, and highest in older people.^6^ Suicidal ideation is similarly associated with all-cause mortality in older people.^7^ Self-harm and suicidal ideation are not themselves diagnoses, but they are associated with a range of mental health problems, with a strong relationship between depression and dying by suicide.^8^ Suicidal ideation is common among people with diagnoses of depressive disorders: with rates of 10 to 48% reported in the scientific literature.^9^

Depression is a common mental disorder in later life. In Ireland, the Irish Longitudinal Study on Ageing (TILDA), a longitudinal cohort study, reported a point prevalence of 10% for clinically significant depressive symptoms in an Irish community-dwelling population aged 50 years and over.^10^ Depression is especially common in people in long-term care and hospitals, where the point prevalence rates range from 17 to 35%.^11,12^ Although much of the research has focused on the association of depression in these settings with quality of life,^13^ it has been reported that the suicide rate steadily increases with age among people over the age of 60, and there is evidence that rates of suicide *attempts* steadily reduce with age. Hawton and Harris, in their study of suicide over the lifespan, found that the ratio of self-harm to suicide varied from 10:1 in individuals aged 60 and older to 200:1 among teenagers.^14^ Even among older adults who died by suicide, the prevalence of self-harm declined with age.

## Suicide and its risk factors in older people

In almost all countries older adults have the highest suicide rate, although self-harm is more frequent among young adults and adolescents. This rate is as high as 48.7/100 000 for older men in the USA (over four times the national age-adjusted rate of 11.1/100 000) and 140/100 000 for older men in rural China.^15^ Definition of ‘older people’ can vary somewhat between countries. Older people are defined as aged 65 and over in studies from the USA and 60 and over in China.^15,16^ These studies reported that the prevalence of social stressors and treatment of any psychiatric disorder decreased with age. In contrast, physical health conditions and bereavement were found to increase with age.

In addition to depression, other risk factors for suicidal death in older adults include being widowed, having other mental illnesses, physical illnesses and bereavement.^17^ The 2019 systematic review by Troya et al identified that physical problems are common in older people who present with self-harm, as is increased loneliness, and perceived burdensome ageing.^18^ Likewise, increased risk of repetition has been reported among older adults with a history of self-harm, and with previous and current psychiatric treatment.^18^ Sinyor et al identified three clusters among adults over the age of 80 who died by suicide: the first cluster comprised those who were married or widowed, the second comprised individuals who were living alone or socially isolated and the third comprised those with a diagnosis of dementia or depression. Fässberg et al in their systematic review reported that physical disability and a range of physical illnesses were significantly associated with suicidality in older people.^19^ The authors reported that social factors such as isolation or reduced connectedness are associated with suicidal ideation, self-harm and death by suicide in older adults.^19^

## Depression and suicidal behaviours in older people

While in Ireland the TILDA study has provided a valuable context for the prevalence of depressive symptoms in older adults, to date, there is only one major study of self-harm in older adults in Ireland. It reported a slightly different pattern to international studies, with lower rates of suicide in older people than those reported in international studies.^20^ More recently, in 2024, Troya et al reported higher rates of suicidal ideation in older people during the years of the COVID-19 pandemic.^21^

Given the high risk of suicide in older adults, identifying the characteristics of self-harm in this population is a clinical priority. Additionally, understanding the clinical, social, geographical and service-related factors associated with suicidal ideations and behaviours is crucial for developing treatments and services that effectively address their needs.

Since older adults have higher rates of death by suicide, and self-harm is a significant risk factor for suicide, examining these variables can help us better identify those at highest risk, including individuals making high-lethality attempts. This information will inform service development and highlight areas where needs are not being adequately met.

## Objectives

The primary objective of this research project is to identify the clinical and sociodemographic variables associated with psychiatric admission among older adults who present with self-harm and with suicidal ideation to emergency departments. We also aimed to examine diagnostic profiles and differences between those aged over 60 years presenting with self-harm and those with suicidal ideation.

## Methods

This is a cohort study of all patients presenting to Irish emergency departments with self-harm or suicidal ideation aged 60 and over. We utilised the national data-set held by the National Clinical Programme for Self-Harm and Suicide-Related Ideation (NCPSHI) in the Health Service Executive (HSE: the state agency tasked with the provision of health and social care in Ireland). This programme falls under the governance of the National Clinical Programmes for Mental Health and was established in 2010 as a collaborative initiative between the HSE Clinical Strategy and Programmes Division and the College of Psychiatrists of Ireland. In the same year, a working group developed the NCPSHI to ensure that all patients who self-harm or have suicidal ideation and present to the emergency department receive a timely, expert biopsychosocial assessment.^22^ The NCPSHI manages the assessment and treatment of self-harm and suicidal ideation in acute hospital emergency departments.

Since 2018, a database has been maintained which includes the demographic and clinical characteristics of people presenting with self-harm and suicidal ideation to emergency departments across Ireland. This national data-set encompasses the years 2018–2022, comprising a 5-year cohort with a total of 72 810 presentations across all age groups. Data on all presentations and clinical activities within the NCPSHI are collected and centrally collated, and this database is held and administered by the NCPSHI.^22^ This data includes all anonymised presentation data from the participating adult emergency departments (24 of the 26 emergency departments in Ireland).

### The data-set

This data-set is completely anonymised at point of data-entry. The NCPSHI provides the following suicide interventions, which are recorded in the data-set for each presentation: biopsychosocial assessment, letter to general practitioner (GP) sent within 24 h, patient given a collaboratively developed Emergency Safety Plan, collateral history obtained and next of kin involvement. Follow-up calls for those assessed within 24 h of assessment are also recorded. In addition to their clinical role of conducting assessments in the emergency department, the Clinical Nurse Specialists (CNS) of the NCPSHI play a key role in co-ordinating data collection to monitor and evaluate the outcomes of the programme. The NCPSHI also ensures that CNSs and their line managers receive regular training on data collection procedures.

Data collected by the NCPSHI are completely anonymised at the point of entry into the NCPSHI electronic templates. The data-set includes all individuals seen by the liaison psychiatry or on-call psychiatry service, ensuring that data-only presentations are recorded, without any possibility of identifying the individuals themselves. This means that it is not possible to determine whether an individual accounts for multiple presentations.

Sociodemographic information is collected. Since 2018, age group has been recorded under the following age categories <19, 20–29, 30–39, 40–49, 50–59 and >60. Other sociodemographic variables recorded include gender, ethnic background (following the Central Statistics Office ethnicity grouping), employment status (collected since beginning of 2020), hospital and region.

Information is collected regarding the mode of self-harm act and/or type of ideation (suicidal ideation or self-harm ideation), lethality of attempt (collected since beginning of 2020 – this is assessed by the CNS entering the data), substances contributing to the presentation (including alcohol and drugs) and current attendance with a mental health service. Where a person has presented with any form of self-harm, this is recorded as such, regardless of the degree of accompanying ideation. Suicidal ideation is used for people with thoughts of suicide or self-harm, but who have not harmed themselves at this visit. Additional information is recorded on the source of referral: self/family/supportive friend, GP, police, mental health services, voluntary organisations or another source. Details are also recorded regarding referral onwards, including admission to hospital and referral to a range of statutory or voluntary agencies.

### Power calculation

Our study was a retrospective cohort analysis of all presentations to the national service (*n* = 72 810 over 5 years) therefore a power calculation was not required. Older people represented a subgroup (*n* = 5041; 6.9%) of the total data-set.

### Statistical analysis

We examined the characteristics of suicidality in our population, using descriptive analysis. We used chi-square tests for bivariate analysis: this permitted some comparisons between the frequencies of variables between the groups with and without self-harm. We explored clinical and demographic variables associated with suicidal ideation and behaviours using multivariable analysis, to allow us to control for variables including gender, substance use, history of contact with mental health services and admission to hospital. The significance level was set at 0.05 and SPSS software was used (version 29 for macOS; available at: https://www.ibm.com/products/spss-statistics). We conducted subgroup analyses examining the differences between clinical variables associated with self-harm and suicidal ideation in older adults nationally from 2018 to 2022. We examined the relationship between self-harm and suicidal ideation and psychiatric admission using logistic regression and controlled for gender, year of presentation, comorbid substance use and medical admission.

## Results

Our data-set comprised 72 810 presentations of all people presenting with self-harm and/or suicidal ideation to 24 emergency departments in Ireland from January 2018 to December 2022. Of these, 3487 presentations did not include the minimum fields required for analysis and were excluded, leaving 69 383 for analysis. The majority of those presentations were of people aged under 60 (*n* = 64 517), with 4866 (7%) presentations of people aged over 60 years. People aged over 60 years made up 6.1% (*n* = 2193/35 734) of presentations with self-harm and 7.9% (*n* = 2673/33 649) of those presenting with suicidal ideation (see Table 1).

**Table 1:** Sociodemographic characteristics of people presenting with self-harm compared with those presenting with suicidal ideation Table 1 long description.

Characteristics	Total, *n* (%)	Self-harm, *n* (%)	Suicidal ideation, *n* (%)	χ^2^	*p*-value
	(*n* = 69 383)	(*n* = 35 734)	(*n* = 33 649)		
Age					
<19	9899 (14.3)	6084 (17.0)	3805 (11.3)	555.13	<0.001
20–29	20 379 (29.4)	10 593 (29.7)	9786 (29.1)		
30–39	14 836 (21.4)	7301 (20.5)	7535 (22.4)		
40–49	11 754 (17.0)	5849 (16.4)	5905 (17.6)		
50–59	7596 (11.0)	3675 (10.3)	3921 (11.7)		
60 years and over	4866 (7.0)	2193 (6.1)	2673 (7.9)		
Gender					
Female	35 439 (51.1)	20 488 (57.3)	14951 (44.4)	1165.7	<0.001
Male	33 818 (48.7)	15 177 (42.5)	18 641 (55.4)		
Other/non-binary	121 (0.2)	69 (0.2)	52 (0.2)		
Not recorded	<10	<10	<10		
Ethnic background					
Asian	304 (0.4)	156 (0.4)	150 (0.4)	75.1	<0.001
Black	228 (0.6)	291 (0.9)	519 (0.8)		
White Irish	30 757 (86.1)	28 835 (85.7)	59 592 (85.9)		
Irish Traveller	1171 (3.3)	988 (2.9)	2159 (3.1)		
Other/White	2045 (5.7)	1762 (5.2)	3807 (5.5)		
Other	1377 (3.9)	1623 (4.9)	3000 (4.3)		
Employment status					
Working	4998 (14)	5135 (15.3)	10 133 (14.6)	300.9	<0.001
Not working	14 274 (39.9)	14 281 (42.4)	28 565 (41.2)		
Unknown	16 462 (46.1)	14 223 (42.3)	30 685 (44.2)		
Year					
2018	12 589 (18.3)	6882 (19.3)	5707 (17.0)	–	–
2019	12 553 (18.1)	6842 (19.1)	5711 (17.0)		
2020	14 490 (20.9)	7729 (21.6)	6761 (20.1)		
2021	13 882 (20.0)	6776 (19.0)	7106 (21.1)		
2022	15 869 (22.9)	7505 (21.0)	8364 (24.9)		

In the group aged over 60 years as a whole, there were similar proportions of men (*n* = 2469, 50.7%) and women. However, there were higher proportions of older women presenting with self-harm (58.4%) compared with men (41.5%), and higher proportions of men presenting with suicidal ideation (58.3%) compared with women; and these differences were statistically significant (χ^2^ = 136.7; *p* < 0.001 see Table 2).

**Table 2:** Sociodemographic characteristics of people aged 60 and over presenting with self-harm compared with those presenting with suicidal ideation Table 2 long description.

Characteristics	Total, *n* (%) (*n* = 4866)	Self-harm, *n* (%) (*n* = 2193)	Suicidal ideation, *n* (%) (*n* = 2673)	χ^2^	*p*-value
Gender					
Female	2394 (49.2)	1281 (58.4)	1113 (41.6)	136.7	<0.001
Male	2469 (50.7)	910 (41.5)	1559 (58.3)		
Other/non-binary	<10	<10	<10		
Ethnic background					
Asian	<10	<10	<10	12.08	0.06
Black	<10	<10	<10		
White Irish	4480 (92.1)	2031 (92.6)	2449 (91.6)		
Irish Traveller	53 (1.1)	28 (1.3)	25 (0.9)		
Other/White	182 (3.7)	83 (3.8)	99 (3.7)		
Other	139 (2.8)	49 (2.2)	90 (3.4)		
Employment status					
Working	315 (6.5)	140 (6.4)	175 (6.5)	34.85	<0.001
Not working	2616 (53.7)	1088 (38.8)	1528 (57.2)		
Unknown	1935 (39.8)	965 (49.8)	970 (36.3)		
Year					
2018	811 (16.7)	409 (18.7)	402 (15.0)	–	–
2019	812 (16.7)	413 (18.8)	399 (14.9)		
2020	919 (18.9)	439 (20)	480 (18.0)		
2021	1047 (21.5)	404 (18.4)	643 (24.1)		
2022	1277 (26.2)	528 (24.1)	749 (28.0)		

Consistent with the methodology of the National Clinical Programme for Self-Harm and Suicide-Related Ideation, any field with fewer than ten presentations is presented as <10.

In terms of ethnicity, White Irish people made up the vast majority of presentations in both self-harm and suicidal ideation groups aged over 60 years; 92.6. and 91.6% respectively (*p* = 0.06). People of Asian or Black ethnicities represented less than 1% of the total presentation (Table 2). Irish Travellers represented 3.1% of presentations across the whole population presenting with self-harm and suicidal ideation, but only 1.1% of the older population.

As shown in Table 3, the majority of presentations of people aged > 60 years were assessed in the emergency department (80%). Those people who presented with self-harm were significantly more likely to be assessed on a medical/surgical ward or in an intensive care unit (29.3%, <0.001) than those without self-harm (12.4%, <0.001).

**Table 3:** Clinical characteristics of people aged 60 and over presenting with self-harm compared with those presenting with suicidal ideation Table 3 long description.

Characteristics	Total, *n* (%) (*n* = 4866)	Self-harm, *n* (%) (*n* = 2193)	Suicidal ideation, *n* (%) (*n* = 2673)	χ^2^	*p*-value
Substance use					
Alcohol and drugs	90 (1.8)	57 (2.6)	33 (1.2)	41.79	<0.001
Alcohol only	1327 (27.3)	601 (27.4)	726 (27.2)		
Drugs only	76 (1.6)	57 (2.6)	19 (0.7)		
Nil	3373 (69.3)	1478 (67.4)	1895 (70.9)		
Attending mental health team					
No/unknown	2928 (61.7)	1328 (62.2)	1600 (61.3)	0.36	0.551
Yes	1817 (38.3)	808 (37.8)	1009 (38.7)		
Referred to emergency department by					
Mental health team	73 (1.5)	27 (1.3)	46 (1.7)	237.1	<0.001
General practitioner	698 (14.4)	137 (6.3)	561 (21.0)		
Police	162 (3.3)	59 (2.7)	103 (3.9)		
Other	3926 (80.8)	1967 (89.7)	1959 (73.4)		
Assessment location					
Emergency department	3734 (80.0)	1492 (70.7)	2242 (87.6)	205.5	<0.001
Medical/surgical/intensive care unit	934 (20.0)	617 (29.3)	317 (12.4)		
Received assessment					
Assessed	4668 (95.9)	2109 (96.2)	3559 (95.7)	0.58	0.445
Not assessed	198 (4.1)	84 (3.8)	124 (4.3)		
Subsequent admission to psychiatry					
No	3520 (75.4)	1543 (73.2)	1977 (77.3)	10.2	<0.001
Yes	1147 (24.6)	565 (26.8)	1582 (22.7)		
Next referrals					
Specialist mental health service	1917 (54.7)	833 (54.5)	1084 (54.9)	0.74	0.785
Other	1585 (45.3)	696 (45.5)	889 (45.1)		
Time to admission/discharge					
<6 h	2743 (62.1)	1058 (53.8)	1685 (68.7)	106.9	<0.001
<9 h	707 (16.0)	361 (18.4)	346 (14.1)		
>9 h	968 (21.9)	546 (27.8)	422 (17.2)		

While across all ages, the majority of patients were not admitted to a psychiatric ward (*n* = 53 928, 83.2%), one-quarter (25%) of those aged over 60 were. In the older population, those presenting with self-harm had a higher proportion of admissions than those with suicidal ideation (26.8 *v*. 22.7%, *p* < 0.001), and higher than the all-age admission rate in those presenting with self-harm (16.3%).

In older people, overdose was the most common method of self-harm, representing 71.6% of all self-harm presentations (*n* = 1571). Cutting was the second most common, totalling 10.8% of self-harm presentations (*n* = 237). Of those whose lethality was assessed, one-fifth (20.3%) were deemed to be high lethality (see Table 4).

**Table 4:** Characteristics of self-harm in the population aged over 60 Table 4 long description.

Characteristics of self-harm	Aged >60 years, *n* (%) (*n* = 2193)
Type of self harm	
Cutting	237 (10.8)
Attempted drowning	72 (3.3)
Drug overdose	1571 (71.6)
Attempted hanging	66 (3.0)
Jumping from a height	20 (0.9)
Shooting	3 (0.1)
Multiple methods	55 (2.5)
Attempted suffocation	1 (0.1)
Other	168 (7.7)
Lethality of attempt (only coded 2020–2022, *n* = 1072)	
Low	417 (38.9)
Moderate	437 (40.8)
High	218 (20.3)

Over one-third of people aged over 60 who presented with self-harm were already attending a mental health service (37.8%), similar to those with suicidal ideation (38.7%); this difference was not statistically significant. It is noteworthy that the majority of presentations with self-harm were not currently attending a mental health service (61.7%). Time to admission or discharge from the emergency department is recorded by the NCPSHI in the following categories: less than 6 h, less than 9 h and more than 9 h (see Table 3). Presentations with self-harm were significantly more likely to wait more than 9 h for admission/discharge (27.8 *v*. 17.2%, *p* < 0.001). Those with suicidal ideation were more likely to be admitted or discharged in less than 6 h (68.7 *v*. 53.8%, *p* < 0.001).

On multivariable analysis, the relationship between self-harm and psychiatric admission was explored (with suicidal ideation as the reference group). On a simple logistic regression model (model 1) there was an odds ratio of 1.07 (95% CI 1.028–1.116) for the relationship between self-harm and subsequent admission in those patients aged 60 years and over. This demonstrated that people presenting with self-harm are 1.07 times more likely to require a psychiatric admission (Table 5). In model 2, which controlled for gender, year of presentation, substance use and need for medical admission, the association was no longer statistically significant with an odds ratio 0.979 (95% CI 0.938–1.021). In this model, male gender, absence of substance use and need for medical admission were all significantly associated with need for psychiatric admission. This suggests that people presenting with self-harm are not more likely to require a psychiatric admission than those presenting with suicidal ideation, after controlling for the other variables.

**Table 5:** Logistic regression examining the relationship between self-harm and subsequent psychiatric admission in the population aged over 60 (reference population are those presenting with suicidal ideation) Table 5 long description.

Regression models	Odds ratio	*p*-value	Confidence interval
Model 1			
Self-harm	1.07	0.001	1.028–1.116
Model 2			
Self-harm	0.979	0.936	0.938–1.021
Gender^a^	1.344	<0.001	1.287–1.402
Substance use	0.587	<0.001	0.562–0.613
Need for medical admission	0.846	<0.001	0.787–0.911

a. For gender, an odds ratio >1 indicates higher odds for males.

## Discussion

This study examined the characteristics of people aged over 60 who presented following self-harm and suicidal ideation. It found that self-harm was significantly associated with admission to a psychiatric facility, but not after controlling for gender, substance use and need for medical admission, and these covariates remained significantly associated with subsequent psychiatric admission in this model.

We found that poisoning or overdose is the most common form of self-harm in older people. Troya et al reported a similar pattern in their 2019 systematic review (68.1%), and similarly, lacerations were the second most common method of self-harm.^18^

The clinical programme has reported overall psychiatric admission rates of 16.8% of the population as a whole undergoing assessment in the emergency department following a presentation with self-harm or suicidal ideation. The admission rates reported in older people are significantly higher for both those presenting with self-harm and suicidal ideation, with the highest rates in older people presenting with self-harm (26.8%). This database does not collect diagnoses, but given that psychiatric admission is a rare outcome reserved for those with more severe mental illness, which is decided only after a detailed mental health assessment and discussion with a consultant psychiatrist, it may be considered a proxy measure for severe mental illness.^23,24^ While depression is the illness more commonly associated with suicidal ideations and behaviours, self-harm and suicidal ideations can also be seen in a range of conditions from psychosis to adjustment disorders.^25,26^ These results suggest that self-harm is not more likely than suicidal ideation to result in psychiatric admission once other variables are controlled for, and that both presentations (self-harm and suicidal ideation) are associated with a need for admission to a similar extent.

Our findings suggest that there are significant differences between older patients who present to the emergency department with self-harm, compared with those presenting with suicidal ideation. Regarding the relationship between self-harm in older people and gender, we found that there were higher proportions of older women presenting with self-harm (58.4%) compared with men (41.5%), and higher proportions of men presenting with suicidal ideation (58.3%) compared with women. This is consistent with the international literature as summarised in the systematic review by Troya et al, which found that among older people, more women than men presented with self-harm.^18^ This is at variance with the higher suicide rate in older men, and it suggests that while more women engage in self-harm, there may be a higher burden of illness associated with self-harm in older men.

The relatively low proportion of older patients who presented with self-harm or suicidal ideation who were previously known to mental health services is surprising. However, it is consistent with Szücs et al’s finding that an older age of onset of mental illness is associated with suicidal behaviour in older people.^27^ It suggests that this population may present to hospital in a suicidal crisis, rather than to their GPs at an earlier stage, or perhaps the crisis evolves more rapidly. It is likewise consistent with the findings of the study by Ho et al, which similarly reported that 37.5% of older people had a history of mental illness, in their study of older people who died by suicide in Singapore.^28^ They reported that despite the mental illness rate of 37.5%, only 17% had an antidepressant detected on toxicology, which suggests that perhaps adherence to medication was suboptimal.^28^ The majority of older people stayed for assessment, and this was higher in those presenting with self-harm, perhaps due to the urgency of medical treatment.

Alternatively, the lower rates of referrals via GPs suggest that access to GPs has become more limited for this population. It raises the question of whether this may have been due in part to COVID restrictions and reduced in-person visits to GPs, so that the emergency department was perceived as the only option.^21,29^ There is an international move towards the digital provision of care.^30,31^ It has been identified in the literature that the indiscriminate use of digital solutions may inadvertently exclude older patients.^32^

Similarly, medical admission can be seen as a proxy and objective measure for the medical lethality of self-harm. This is consistent with the existing literature which suggests that an older age of onset of symptoms is associated with higher lethality of self-harm.^27^ If we consider suicidality as a spectrum, it might be seen to extend from suicidal thoughts, through acts with death by suicide as the most severe end of this clinical spectrum. Those patients who require medical admission for self-harm are likely to represent the more severe end of the spectrum than those who can be medically discharged from the emergency department.^33^ Existing research suggests that in addition to having high suicide rates, older people are more likely to die following self-harm. This is thought to be related to the use of lethal means, a greater degree of planning (less impulsivity) and physical frailty^34^ (as represented by the need for medical admission as a proxy measure).

Substance use, in this study, was associated with self-harm in older people, and there were similar proportions of people using alcohol among those who presented with self-harm and suicidal ideation. There were significant differences, however, in the use of other substances, with higher proportions of those with self-harm using either drugs and alcohol, or just drugs. Ho et al reported that alcohol was a feature in older people dying by suicide in 23.1% of cases, which is slightly lower than our cohort.^28^ That said, the majority of older patients presenting with self-harm and suicidal ideation did not use substances at all, with a higher proportion of those presenting with self-harm using substances, with or without alcohol.

Finally, older people attending the emergency department with suicidal ideation had longer waiting times in the emergency department than those attending with self-harm. This finding is somewhat counterintuitive: we expected that those presenting with self-harm, who likely require medical/surgical input for injuries or poisoning, would be longer in the emergency department, awaiting bloods and a range of clinical opinions. This is important from a service perspective, as the emergency department is a high-stimulus environment which can present challenges for older persons, or indeed anyone experiencing acutely distressing symptoms.

### Strengths and limitations

One of the strengths of this study is the utilisation of a national data source of self-harm and suicidal ideation to identify older adults who present with self-harm and suicidal ideation. It includes some clinical information and allows for comparisons to be made between people presenting with self-harm and suicidal ideation across different age profiles. This is the first examination of this data in older people utilising an Irish sample. As an Irish sample, the findings may not be generalisable to other countries.

The retrospective nature of the evaluation limits the study; however, the population studied is a complete clinical sample. It is further limited in that the national data source does not include clinical diagnosis and is presentation-based rather than individual-based, and therefore, repeat presentations of individuals are not captured. This means that the data-set includes individuals with multiple presentations, which may have an impact on the independence assumption of the logistic regression model, and risks bias through incorrect inferences.

### Implications

This study raises questions such as the role of psychiatric diagnosis in the consideration of suicidal risk in this population, and further work might examine psychiatric diagnosis in a population presenting with self-harm and suicidal ideation. Furthermore, there is a need for a qualitative assessment to examine the experiences of older people attending emergency departments with suicidal ideation or self-harm: at present, there is limited research in this area.^35^ This would help to consider the motivations and the themes which underlie self-harm in this vulnerable and high-risk population. This study adds evidence in an Irish context of the importance of acknowledging the high morbidity and risk associated with older people presenting with self-harm and suicidal ideation. Further prospective research in this area could inform future practice.

The results of this study demonstrate the significant differences in characteristics of older people presenting to Irish emergency departments with self-harm compared with suicidal ideation, where previously a paucity of data existed. The association with needs for both mental and physical healthcare identified among older people makes it imperative to identify the characteristics of self-harm in this population. This would help us to understand the factors associated with increased risk and to develop treatments and services to meet their needs. The number of presentations among older people makes it imperative to understand the factors driving these presentations, and to tailor our services to meet the needs of older people presenting to emergency departments for care.

## Data Availability

The data are not publicly available due to the data-set being primarily the property of the National Clinical Programme for Self-Harm and Suicide-Related Ideation, Health Service Executive, Ireland.

## Table 1 Long description

A table comparing sociodemographic characteristics of people presenting with self-harm and suicidal ideation. The table has 6 rows and 10 columns. Column headers are Total, Self-harm, Suicidal ideation, χ2, and P value. Row labels are Age, Gender, Ethnic background, Employment status, and Year. Row 1: Age, Total (n = 69,383), Self-harm (n = 35,734), Suicidal ideation (n = 33,649), χ2, P value. Row 2: <19, 9899 (14.3), 6084 (17.0), 3805 (11.3), 555.13, <0.001. Row 3: 20-29, 20379 (29.4), 10593 (29.7), 9786 (29.1). Row 4: 30-39, 14836 (21.4), 7301 (20.5), 7535 (22.4). Row 5: 40-49, 11754 (17.0), 5849 (16.4), 5905 (17.6). Row 6: 50-59, 7596 (11.0), 3675 (10.3), 3921 (11.7). Row 7: 60 years and over, 4866 (7.0), 2193 (6.1), 2673 (7.9). Row 8: Gender, Total, Self-harm, Suicidal ideation. Row 9: Female, 35439 (51.1), 20488 (57.3), 14951 (44.4). Row 10: Male, 33818 (48.7), 15177 (42.5), 18641 (55.4). Row 11: Other/non-binary, 121 (0.2), 69 (0.2), 52 (0.2). Row 12: Not recorded, <10, <10, <10. Row 13: Ethnic background, Total, Self-harm, Suicidal ideation. Row 14: Asian, 304 (0.4), 156 (0.4), 150 (0.4). Row 15: Black, 228 (0.6), 291 (0.9), 519 (0.8). Row 16: White Irish, 30757 (86.1), 28835 (85.7), 59592 (85.9). Row 17: Irish Traveller, 1711 (3.3), 988 (2.9), 2159 (3.1). Row 18: Other/Whiteee, 2045 (5.7), 1762 (5.2), 3807 (5.5). Row 19: Other, 1377 (3.9), 1623 (4.9), 3000 (4.3). Row 20: Employment status, Total, Self-harm, Suicidal ideation. Row 21: Working, 4998 (14), 5135 (15.3), 10133 (14.6). Row 22: Not working, 14274 (39.9), 14281 (42.4), 28565 (41.2). Row 23: Unknown, 16462 (46.1), 14223 (42.3), 30685 (44.2). Row 24: Year, Total, Self-harm, Suicidal ideation. Row 25: 2018, 12589 (18.3), 6882 (19.3), 5707 (17.0). Row 26: 2019, 12553 (18.1), 6842 (19.1), 5711 (17.0). Row 27: 2020, 14490 (20.9), 7729 (21.6), 6761 (20.1). Row 28: 2021, 13882 (20.0), 6776 (19.0), 7106 (21.1). Row 29: 2022, 15869 (22.9), 7505 (21.0), 8364 (24.9).

Navigate back to Table 1.

## Table 2 Long description

A table comparing sociodemographic characteristics of people aged 60 and over with self-harm and suicidal ideation. The table has 4866 total entries, divided into self-harm (2193) and suicidal ideation (2673). It includes columns for gender, ethnic background, employment status, and year. Row 1: Gender, Female, 2394 (49.2), 1281 (58.4), 1113 (41.6). Row 2: Gender, Male, 2469 (50.7), 910 (41.5), 1559 (58.3). Row 3: Gender, Other/non-binary, <10, <10, <10. Row 4: Ethnic background, Asian, <10, <10, <10. Row 5: Ethnic background, Black, <10, <10, <10. Row 6: Ethnic background, White Irish, 4480 (92.1), 2031 (92.6), 2449 (91.6). Row 7: Ethnic background, Irish Traveller, 53 (1.1), 28 (1.3), 25 (0.9). Row 8: Ethnic background, Other/White, 182 (3.7), 83 (3.8), 99 (3.7). Row 9: Ethnic background, Other, 139 (2.8), 49 (2.2), 90 (3.4). Row 10: Employment status, Working, 315 (6.5), 140 (6.4), 175 (6.5). Row 11: Employment status, Not working, 2616 (53.7), 1088 (50.8), 1528 (57.2). Row 12: Employment status, Unknown, 1935 (39.8), 965 (44.8), 970 (36.3). Row 13: Year, 2018, 811 (16.7), 409 (18.7), 402 (15.0). Row 14: Year, 2019, 812 (16.7), 413 (18.8), 399 (14.9). Row 15: Year, 2020, 919 (18.9), 439 (20), 480 (18.0). Row 16: Year, 2021, 1047 (21.5), 404 (18.4), 643 (24.1). Row 17: Year, 2022, 1277 (26.2), 528 (24.1), 749 (28.0).

Navigate back to Table 2.

## Table 3 Long description

A table comparing clinical characteristics of people aged 60 and over presenting with self-harm and suicidal ideation. The table has 11 rows and 6 columns. Column headers are Total (n = 4866), Self-harm (n = 2193), Suicidal ideation (n = 2673), χ2, and P value. Row labels include Substance use, Attending mental health team, Referred to Emergency Department by, Assessment location, Received assessment, Subsequent admission to psychiatry, Next referrals, and Time to admission/discharge. Each row provides specific data points for each category. Notable trends include higher percentages of alcohol use and emergency department assessments among those presenting with self-harm compared to those with suicidal ideation.

Navigate back to Table 3.

## Table 4 Long description

A table showing characteristics of self-harm in individuals aged over 60. The table has 11 rows and 3 columns. The columns are labeled Type of self harm, and Lethality of attempt (only coded 2020-2022). The table presents data for individuals aged over 60 years (n=2193). Row 1: Cutting, 237 (10.8 percent). Row 2: Attempted drowning, 72 (3.3 percent). Row 3: Drug overdose, 1571 (71.6 percent). Row 4: Attempted hanging, 66 (3.0 percent). Row 5: Jumping from a height, 20 (0.9 percent). Row 6: Shooting, 3 (0.1 percent). Row 7: Multiple methods, 55 (2.5 percent). Row 8: Attempted suffocation, 1 (0.1 percent). Row 9: Other, 168 (7.7 percent). Row 10: Low lethality, 417 (38.9 percent). Row 11: Moderate lethality, 437 (40.8 percent). Row 12: High lethality, 218 (20.3 percent).

Navigate back to Table 4.

## Table 5 Long description

A table with three columns and six rows, including a header row. The columns are labeled ‘Model’, ‘Odds ratio’, ‘p-value’, and ‘Confidence interval’. The table presents data from two models examining the relationship between self-harm and psychiatric admission in individuals aged over 60. Model 1 shows an odds ratio of 1.07 with a p-value of 0.001 and a confidence interval of 1.028-1.116. Model 2 includes additional variables: gender, substance use, and need for medical admission. The odds ratio for self-harm in Model 2 is 0.979 with a p-value of 0.936 and a confidence interval of 0.938-1.021. Other variables in Model 2 include gender with an odds ratio of 1.344, substance use with an odds ratio of 0.587, and need for medical admission with an odds ratio of 0.846, all having p-values of less than 0.001.

Navigate back to Table 5.
